# Daily adaptive radiotherapy for patients with prostate cancer using a high field MR-linac: Initial clinical experiences and assessment of delivered doses compared to a C-arm linac

**DOI:** 10.1016/j.ctro.2020.04.011

**Published:** 2020-04-29

**Authors:** Alex Dunlop, Adam Mitchell, Alison Tree, Helen Barnes, Lorna Bower, Joan Chick, Edmund Goodwin, Trina Herbert, Rebekah Lawes, Helen McNair, Dualta McQuaid, Jonathan Mohajer, Rahul Nilawar, Angela Pathmanathan, Gillian Smith, Ian Hanson, Simeon Nill, Uwe Oelfke

**Affiliations:** a The Joint Department of Physics, the Royal Marsden Hospital and the Institute of Cancer Research, United Kingdom; b The Royal Marsden NHS Foundation Trust and the Institute of Cancer Research, United Kingdom; c The Institute of Cancer Research, United Kingdom; d The Royal Marsden NHS Foundation Trust, United Kingdom

**Keywords:** Adaptive radiotherapy, Prostate cancer, MR-guided radiotherapy, MR-linac, Interfractional motion, High-field MR-linac

## Abstract

**Introduction:**

MR-guided adapted radiotherapy (MRgART) using a high field MR-linac has recently become available. We report the estimated delivered fractional dose of the first five prostate cancer patients treated at our centre using MRgART and compare this to C-Arm linac daily Image Guided Radiotherapy (IGRT).

**Methods:**

Patients were treated using adapted treatment plans shaped to their daily anatomy. The treatments were recalculated on an MR image acquired immediately prior to treatment delivery in order to estimate the delivered fractional dose. C-arm linac non-adapted VMAT treatment plans were recalculated on the same MR images to estimate the fractional dose that would have been delivered using conventional radiotherapy techniques using a daily IGRT protocol.

**Results:**

95% and 93% of mandatory target coverage objectives and organ at risk dose constraints were achieved by MRgART and C-arm linac delivered dose estimates, respectively. Both delivery techniques were estimated to have achieved 98% of mandatory Organ At Risk (OAR) dose constraints whereas for the target clinical goals, 86% and 80% were achieved by MRgART and C-arm linac delivered dose estimates.

**Conclusions:**

Prostate MRgART can be delivered using the a high field MR-linac. Radiotherapy performed on a C-arm linac offers a good solution for prostate cancer patients who present with favourable anatomy at the time of reference imaging and demonstrate stable anatomy throughout the course of their treatment. For patients with critical OARs abutting target volumes on their reference image we have demonstrated the potential for a target dose coverage improvement for MRgART compared to C-arm linac treatment.

## Highlights

• MR-guided radiotherapy for prostate cancer can be delivered using a high filed MR-linac.
• Comparison of estimated delivered dose between MR-guided adapted radiotherapy and conventional C-arm techniques.
• MR-guided adaptive radiotherapy demonstrates improved target coverage for a subset of prostate cancer patients.

## 1 Introduction

The Elekta Unity MR-linac (MRL) (Elekta AB, Stockhom, Sweden) combines a high field 1.5 T Philips Magnetic Resonance (MR) scanner (Philips Medical Systems, Best, The Netherlands) and a 7MV FFF Elekta linear accelerator [1], [2], [3]. The superior soft-tissue contrast [4], [5], when compared to Computed Tomography (CT) and Cone Beam CT (CBCT), available with the MRL should enable improved pre-treatment image verification whilst the unit also facilitates MR-guided adaptive radiotherapy (MRgART) by allowing treatment plans to be designed on the MR image acquired prior to treatment accounting for the current anatomy.

The MRL potentially offers advantages for prostate cancer radiotherapy compared to C-arm linac treatments. The improved visualisation of the patient’s anatomy on MR [4], [5], compared to CT, should improve the delineation accuracy [6] and reduce inter-observer variation of target volume delineation [7]. Substantial interfraction anatomical variation is observed for a subset of prostate cancer patients [7]. The ability to generate MRgART treatment plans on the MRL means the treatment can be shaped to the anatomy of the patient in their treatment position directly prior to treatment [8], [9] as opposed to the anatomical snapshot acquired during simulation imaging in the conventional radiotherapy workflow which could up to two weeks before treatment. Improved target visibility along with the ability to adapt to the daily anatomy may in turn lead to reduced clinical target volume (CTV) to planning target volume (PTV) margins [6] and potentially enable the implementation of more profound hypofractionation for prostate cancer [6], such as five, three, two, or even single fraction regimes, where accurate delivery and dose constraint compliance of every fraction will be essential. The MRL is also able to acquire real-time MR images as the treatment beam is being delivered meaning intrafraction organ motion can be observed and, in the future, be accounted for via gating or tracking techniques [10], [11], [12], [13], [14].

Low field (0.35T) MRgART has been available since 2014 using the MRIdian treatment unit (ViewRay, Inc., CA, US) [15], [16], [17]. Recent studies have shown that 0.35T-based MRgART for prostate cancer resulted in a low incidence of toxicity for stereotactic body radiation therapy (SBRT) [15] and that adaptive planning can be beneficial when considerable interfraction organ motion is observed [16]. MRgART, with diagnostic-quality high field MR images, using the MRL has been in clinical use globally since 2017 [3] and 2018 at our centre. Feasibility of MRgART using the MRL has recently been reported [18] but, to our knowledge, no study has reported a dosimetric comparison between this and standard C-arm linac delivery for prostate cancer.

The PRISM (Prostate Radiotherapy Integrated with Simultaneous MRI; NCT 03658525) trial aims to assess the feasibility, safety, and tolerability of MRL-guided prostate radiotherapy [6]. In this paper we describe the clinical implementation of high-field MR-guided adaptive radiotherapy (MRgART) for patients having MRL prostate radiotherapy within the PRISM trial and evaluate estimates of delivered dose for the first five prostate cancer patients reported to have received daily MRgART using the MRL. We compare these results with estimates of delivered doses with treatment using daily IGRT on a C-arm linac and to our knowledge this represents the first reported dosimetric comparison between MRgART using the MRL and C-arm-based IGRT delivery

## 2 Methods

The first five patients recruited to PRISM were included in this study. The primary ‘prostate CTV’ is defined as the prostate and proximal 1 cm of seminal vesicles with a secondary CTV (‘SV CTV’) consisting of the proximal 2 cm of seminal vesicles exterior to the prostate CTV. The CTV to PTV margins, target coverage requirements, and Organ at Risk (OAR) constraints (Table S1) are the same as for conventional daily-online IGRT on a C-arm linac at our centre (Table S1).

### 2.1 Treatment planning

The MRL has two modes of operation: Adapt-To-Position (ATP) and Adapt-To-Shape (ATS), with both online planning methods requiring an initial reference treatment plan to be generated on a reference image (CT or MR) [9], [19]. Bladder and rectal preparation was followed to ensure patients were treated with a comfortably full bladder throughout treatment and scanned without a distended rectum Reference Planning CT scans were acquired with 1.5 mm slice thickness with patients immobilized using the Combi Fix system (Oncology Systems Ltd, UK). Bladder and rectal preparation was followed to ensure patients were treated with a comfortably full bladder throughout treatment and scanned without a distended rectum. MRL reference plans generated on the reference CT image used a 7-field IMRT simultaneous integrated boost technique to deliver 60 Gy and 48.6 Gy in 20 fractions to the primary (PTV_6000) and elective PTVs (PTV_4860), respectively (see Table S1). Dose was calculated to medium using a 0.3 cm isotropic dose grid and 2% statistical uncertainty per plan. Ten segment shape optimisation (SSO) loops were used to generate the reference plans with a maximum of 60 segments, minimum segment area of 4 cm^2^, and minimum of 3 monitor units per segment allowed. After optimisation, the monitor units were re-scaled so that the primary PTV D50% was 60.0 Gy. VMAT Backup treatment plans for a C-arm Elekta Agility linac were generated in the event the MR-linac was not available for treatment and were of the same quality as our standard non-trial prostate plans. Treatment plans for the MRL (both reference and online) were generated using the Monaco (Elekta AB, Stockholm, Sweden, V5.40.00) treatment planning system (TPS) using the GPUMCD [20], [21] dose calculation algorithm and backup plans were generated using the RayStation TPS (Raysearch Laboratories, Stockholm, Sweden, V8.0.0.61) with a collapsed cone dose calculation algorithm.

ATP treatment consists of acquiring a daily MR image, registering this with the reference image, and then adapting the Multi Leaf Collimator (MLC) leaves according to the translations-only rigid registration. The adapted plan is recalculated on the reference image and therefore does not account for inter-fraction anatomical changes. This methodology is akin to daily IGRT-based corrected treatment on a C-arm linac but with MLC leaf adaption to avoid moving the couch in the confined bore of the MRL. In ATS a deformable image registration is performed between the reference and daily images and contours are propagated to the daily image according to this registration. The propagated contours are edited if necessary according to the daily anatomy and a treatment plan is optimised and calculated on the daily image.

### 2.2 Online plan adaption strategy

**Fig. 1:**
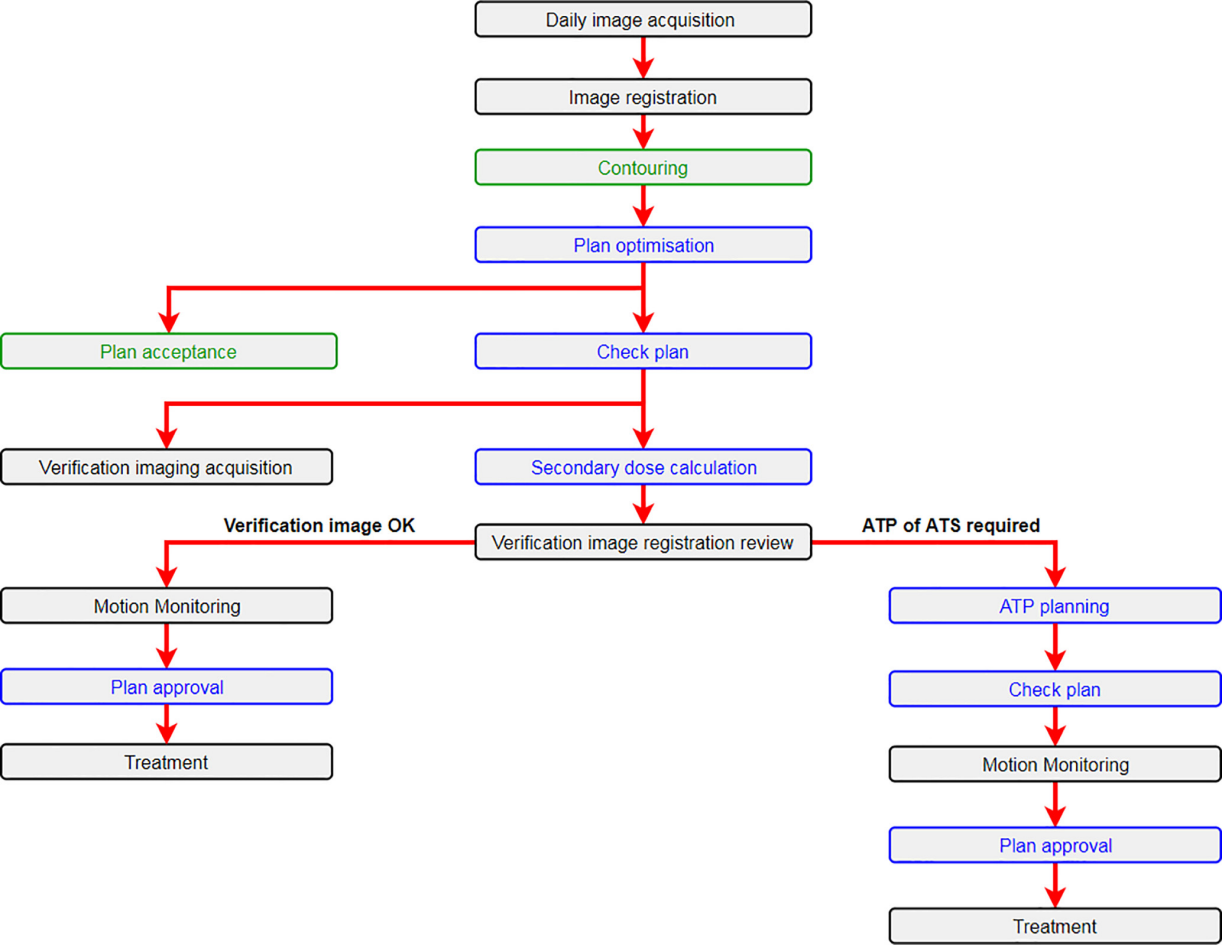
Online MRgART strategy for PRISM patients investigated in this study. Actions in black, green, and blue were performed by radiographers, clinicians, and physicists, respectively. The Motion Monitoring cine MR is acquired throughout entire treatment delivery. (For interpretation of the references to colour in this figure legend, the reader is referred to the web version of this article.)

Fig. 1 illustrates the online MRgART ATS-based workflow implemented to treat PRISM patients at our centre. After a daily ‘online’ plan was generated, checks were carried out to ensure plan parameters were as expected whilst the clinician approved the dose distribution. A secondary dose calculation was then performed using the RayStation TPS and a second MR image was acquired (MR verification image). For online plans, the only modification from the reference plan technique was to perform 5 SSO loops to maintain acceptable online planning times. Dose calculation for the MRgART daily treatment plans was facilitated using a bulk density override method whereby patient-specific overrides were applied to the combined CTVs, bones, and the patient external contour.

If anatomical motion was observed between the time of initial daily MR acquisition and treatment delivery such that the prostate CTV was not completely within the corresponding PTV on the verification image, an ATP of the ATS plan was performed This workflow was implemented from the second patient onwards, for the first case ATS-only plans were generated and delivered.

### 2.3 Delivered fractional dose estimation

In order to estimate the fractional delivered dose, the clinically delivered MRL plans were recalculated on the verification MR image using the same bulk-density override strategy as for the daily image. The verification image and corresponding dose cube were then exported to the RayStation TPS where clinicians contoured target structures (prostate and SV CTVs) and OARs (in order to estimate the delivered fractional dose.

The RayStation TPS-generated VMAT backup plans were used to estimate the fractional dose that would have been delivered on a C-arm linac using a daily online correction IGRT protocol. A soft tissue-based rigid registration was performed by therapeutic radiographers between the reference and daily verification images with the reference image prostate CTV visible thereby mimicking daily online CBCT to CT image matching. The backup plans were then recalculated on each daily verification MR image using the same bulk-density assignment as for the MRL plan calculations. In order to remove systematic differences resulting from the backup and MRL plans being generated in different TPSs with different dose calculation strategies (backup plan generated on the reference CT using a HU-to-density look-up-table and the MRL plans generated using bulk-density assignment), the monitor units of the backup plans were adjusted (average adjustment was −1.5% with standard deviation of 0.2%) so that the primary PTV D50% was 60.0 Gy when the plan was calculated on the reference CT with bulk-density assignments applied as per MRL treatment. Target and OAR dose were then assessed using the clinician-defined verification image ROIs.

### 2.4 Data analysis

Each estimate of fractional dose was scaled to 20 fractions to assess mandatory and optimal clinical goal compliance (Table S1). For the delivered fractional dose estimates the PTV clinical goals were applied to the corresponding CTVs. The dose cubes from the Monaco-generated plans were imported into the RayStation TPS and all dose-volume data were extracted from the RayStation TPS for analysis. All statistical analysis was performed using Python (v2.7.6).

## 3 Results

The MRgART online workflow took 45 min on average. Two fractions were excluded from the analysis due to amended treatment workflows (resulting from hardware and software errors) thus a total of 98 fractions were analysed in this study. Of the 98 analysed fractions, 18 ATP of ATS plans were performed with the remaining 80 fractions being ATS-only workflows.

Table 1 details the results for the mandatory clinical goal dose metrics for the clinical MRL and C-arm linac fractional delivered dose estimates. Of all the assessed mandatory clinical goals, 95% and 93% were achieved by the clinical MRL and C-arm linac delivered dose estimates, respectively. Both delivery techniques were estimated to have achieved 98% of mandatory OAR clinical goals whereas for the target clinical goals, 86% and 80% were achieved by the clinical MRL and C-arm linac delivered dose estimates, respectively.

**Table 1:** Mandatory clinical goal compliance for estimates of delivered fractional dose for five patients treated on the MRL along with corresponding C-arm linac estimates. SD (standard deviation).

			MRL delivered dose			Simulated C-arm linac delivered dose		
ROI	metric	mandatory clinical goal	Mean	SD	% meeting goal	Mean	SD	% meeting goal
Prostate CTV	D99%	n/a	56.2 Gy	2.6 Gy	n/a	55.7 Gy	4.9 Gy	n/a
	D98%	> 55.8 Gy	56.9 Gy	2.2 Gy	83.7	56.8 Gy	3.5 Gy	77.6
	D95%	> 57.0 Gy	57.9 Gy	1.4 Gy	83.7	57.9 Gy	2.5 Gy	81.6
SV CTV	D99%	n/a	49.8 Gy	5.6 Gy	n/a	48.2 Gy	7.5 Gy	n/a
	D98%	> 45.2 Gy	50.6 Gy	5.1 Gy	86.7	48.9 Gy	7.1 Gy	80.6
	D95%	> 46.2 Gy	51.8 Gy	4.6 Gy	88.8	50.2 Gy	6.5 Gy	80.6
Rectum	V60.8 Gy	< 5%	0.6%	1.0%	99.0	0.0%	0.1%	100.0
	V56.8 Gy	< 15%	6.0%	4.5%	95.9	6.5%	3.3%	100.0
	V52.7 Gy	< 30%	12.5%	6.7%	100.0	11.8%	4.8%	100.0
	V48.6 Gy	< 50%	18.7%	8.4%	100.0	16.5%	5.6%	100.0
	V40.5 Gy	< 60%	32.4%	11.3%	100.0	26.5%	7.0%	100.0
Bladder	V60.8 Gy	< 25%	1.2%	1.0%	100.0	0.1%	0.2%	100.0
	V56.8 Gy	< 35%	6.5%	2.8%	100.0	3.0%	1.9%	100.0
	V52.7 Gy	< 50%	10.1%	4.4%	100.0	5.0%	2.9%	100.0
Bowel	V52.7 Gy	< 0.01 cc	0.0 cc	0.2 cc	87.8	0.0 cc	0.2 cc	86.7
	V48.7 Gy	< 6 cc	0.1 cc	0.4 cc	100.0	0.2 cc	0.8 cc	98.9
	D0.01 cc	< 52.7 Gy	36.3 Gy	17.5 Gy	88.8	35.5 Gy	18.4 Gy	86.7
Penile Bulb	V40.5 Gy	< 50%	7.0%	8.7%	100.0	5.3%	7.0%	100.0

**Fig. 2:**
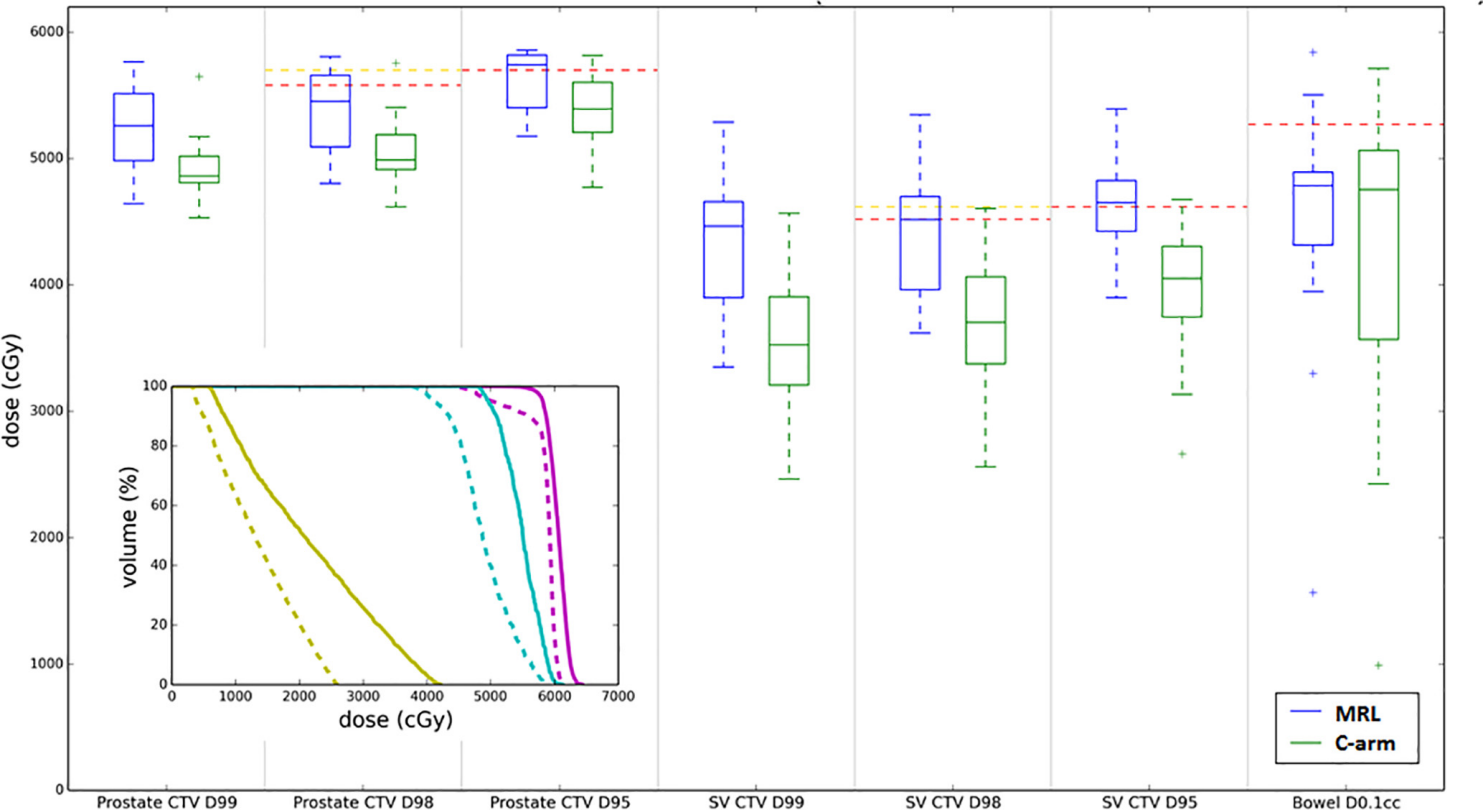
Estimates of delivered dose from case 3. Main figure: boxplots comparing the target coverage dose-volume metrics and critical bowel mandatory maximum dose constraint for the estimates of the clinical MRL and C-arm linac fractional delivered dose estimates. Mandatory and optimal clinical goal levels are shown as red and gold lines, respectively. Inset: an example fractional delivered dose DVH estimate with prostate CTV, SV CTV, and bowel shown as purple, cyan, and yellow, respectively. Clinical MRL and C-arm linac estimates are shown as solid and dashed lines. For both the boxplots and DVHs, all estimates of fractional delivered dose were scaled to 20 fractions. (For interpretation of the references to colour in this figure legend, the reader is referred to the web version of this article.)

**Fig. 3:**
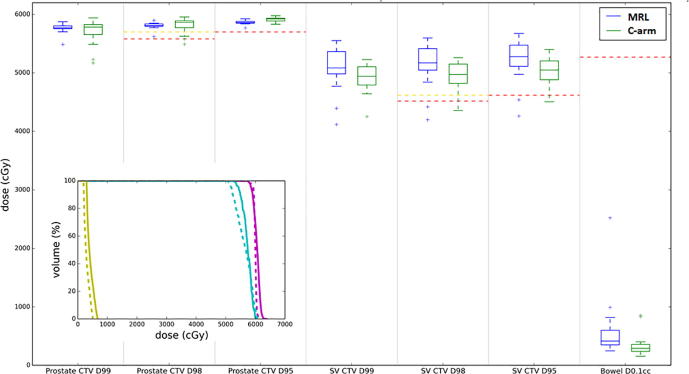
Estimates of delivered dose from case 4. Main figure: boxplots comparing the target coverage dose-volume metrics and critical bowel mandatory maximum dose constraint for the estimates of the clinical MRL and C-arm linac fractional delivered dose estimates. Mandatory and optimal clinical goal levels are shown as red and gold lines, respectively. Inset: an example fractional delivered dose DVH estimate with prostate CTV, SV CTV, and bowel shown as purple, cyan, and yellow, respectively. Clinical MRL and C-arm linac estimates are shown as solid and dashed lines. For both the boxplots and DVHs, all estimates of fractional delivered dose were scaled to 20 fractions. (For interpretation of the references to colour in this figure legend, the reader is referred to the web version of this article.)

**Fig. 4:**
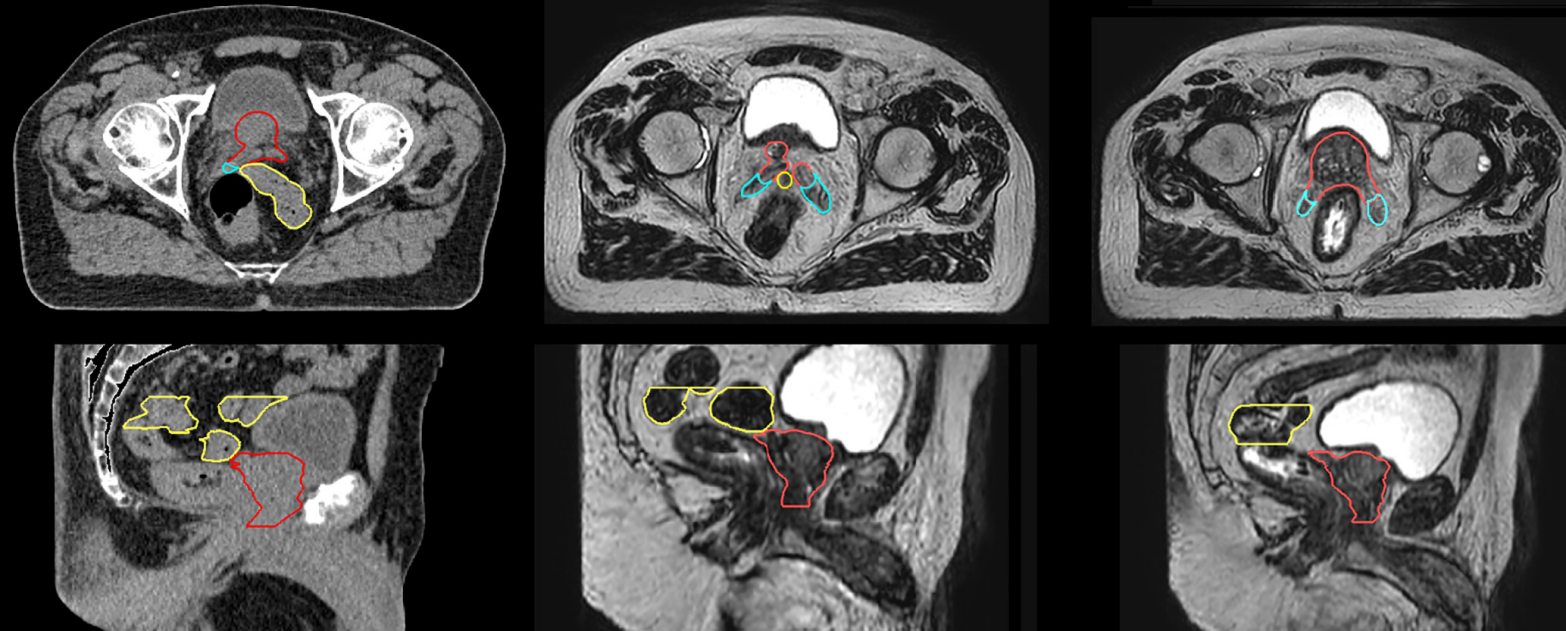
Axial (top row) and sagittal (bottom row) images of case 3 from the planning CT (left column), #5 verification MR acquisition (centre column), and #6 verification MR acquisition (right column). In all images, the prostate CTV (including proximal 1 cm seminal vesicles), SV CTV, and bowel are shown as red, cyan, and yellow contours, respectively. (For interpretation of the references to colour in this figure legend, the reader is referred to the web version of this article.)

Fig. 2 displays clinical MRL and C-arm linac fractional dose estimates for a patient (case 3) who presented with unfavourable anatomy at their reference scan (bowel loop adjacent to the CTV) and exhibited highly variable inter-fraction anatomical motion. Fig. 3 displays the corresponding results for a patient (case 4) with favourable and stable anatomy. For this patient the C-arm linac backup plan and MRL reference plan compromised target coverage to ensure the bowel 52.7 Gy maximum dose constraint was achieved. However, this patient exhibited significant inter-fraction anatomical motion of both the target structures and OARs (Fig. 4). For a proportion of fractions the bowel had moved away from the target volumes meaning the clinically-delivered MRgART plan was able to achieve target coverage requirements (Table S1) on these days. The non-adaptive C-arm linac plans could not account for such inter-fraction changes in anatomy hence target coverage was compromised for all fractions. For the mandatory target coverage clinical goals for case 3, we estimate that with MRgART the prostate CTV D98%, prostate CTV D95%, SV CTV D98%, and SV CTV D95% received on average 54.5 Gy, 57.4 Gy, 45.2 Gy, and 46.5 Gy, respectively with all average results apart from the prostate CTV D98% meeting the mandatory clinical goal. The corresponding estimates for the C-arm linac were 49.9 Gy, 53.9 Gy, 37.0 Gy, and 40.5 Gy, with all averages failing to meet the mandatory clinical goal and representing a range of decreases of 4.6–8.2 Gy compared to the MRgART estimates, equivalent to approximately two additional fractions on the MRL compared to a C-arm linac whilst achieving the same mandatory OAR clinical goal compliance (98% mandatory OAR clinical goal compliance for both MRL and C-arm linac fractional dose estimates). For case 3, 40% of MRL fractional dose estimates achieved the optimal SV CTV clinical goal whereas none of the C-arm linac plans achieved this.

For case 4 (Fig. 3) the patient had bowel far away from target volumes at the time of reference scanning and for every fraction meaning no target compromise was necessary during backup and reference planning. Case 4 was representative of all cases in this study apart from case 3. No significant inter-fraction motion was observed meaning the C-arm linac plan was able to achieve comparable target coverage to the MRgART delivered dose estimates with both techniques estimated to achieve 96% of mandatory target clinical goals.

Out of the 98 comparisons of the prostate CTV D95% metric, the C-arm linac plan achieved a higher CTV D95% compared to the clinical MRgART plan on 71 occasions. The mean CTV prostate D95% out of these 71 cases was 58.8 Gy and 58.0 Gy for the C-arm linac and MRgART estimates, respectively, with both being above the mandatory clinical goal. However, for the remaining 27 cases the mean CTV prostate D95% was 55.2 Gy (<57.0 Gy) and 57.5 Gy for the C-arm linac and MRgART estimates. This indicates that when the C-arm linac plan achieves a higher CTV prostate D95% it is not clinically relevant, whereas for fractions in which the opposite is true the possibility of a clinical impact remains. A corresponding analysis shows similar results for the CTV prostate D98%. Similar to the prostate CTV, for the SV CTV the MRgART achieved the mandatory target coverage clinical goals more often than the C-arm linac plan (Table 1).

**Fig. 5:**
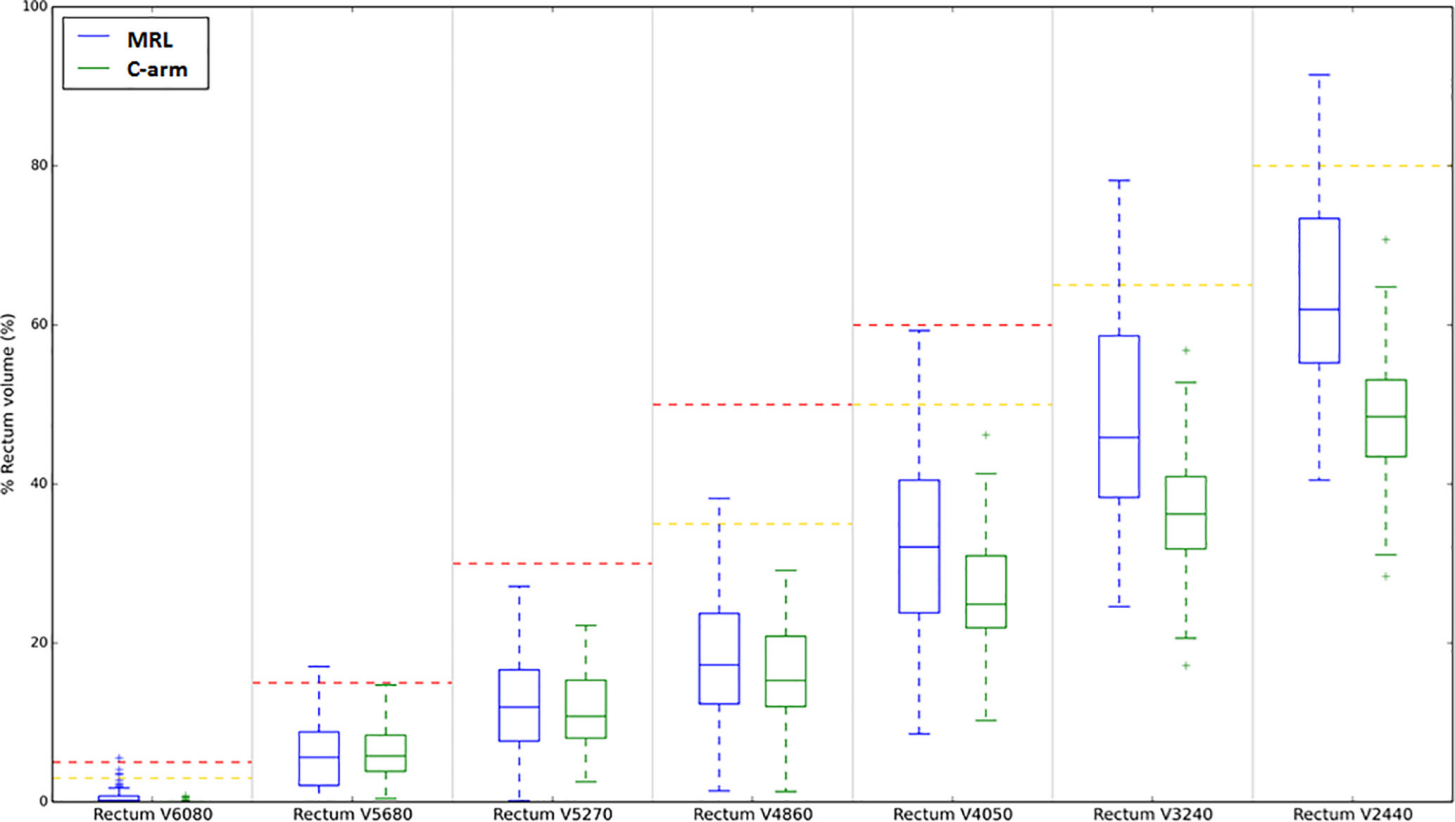
Estimates of delivered rectum dose for all five cases investigated in this study. Boxplots compare rectum dose-volume clinical goal metrics for the estimates of the clinical MRL and C-arm linac fractional delivered dose estimates. Mandatory and optimal clinical goal levels are shown as red and gold lines, respectively. All estimates of fractional delivered dose were scaled to 20 fractions. (For interpretation of the references to colour in this figure legend, the reader is referred to the web version of this article.)

Almost all mandatory OAR clinical goals were achieved for each fractional delivered dose estimate for both the MRgART and C-arm linac plan, however, differences were observed for the rectum V60.8 Gy, low dose rectum, and bladder comparisons and these differences are displayed graphically for all rectum clinical goals in Fig. 5. 79% and 86% of optimal bladder dose constraints were achieved by the clinical MRgART and C-arm linac delivered dose estimates, respectively. The corresponding results for the optimal rectum clinical goals were 91% and 100%. The results for other OARs listed in Table S1 were similar for both delivery techniques.

## 4 Discussion

By estimating delivered fractional doses of MRgART plans we have demonstrated feasibility of prostate treatment on the MRL and, similar to results presented for low-field MRgART [16], the high-field MR guided adapted technique offers a target coverage advantage over a C-arm linac for patients who present with unfavourable anatomy at the time of reference imaging and/or demonstrate significant inter-fraction anatomical variation. We have demonstrated that for patients with unfavourable anatomy, who exhibit an OAR adjacent to the target resulting in target compromise, at the time of reference imaging, MRgART can deliver full target dose for those days when the anatomy is more favourable with critical OARs away from the target volume. Conversely, for patients who present for a fraction with unfavourable anatomy, we have the opportunity to maintain OAR constraint compliance by compromising the target on that day. Such an approach should be undertaken with caution given that C-arm linac radiotherapy, from which OAR clinical goals are based, does not adapt dose distributions and limit target dose in this way and toxicity outcomes are considered acceptable [22]. However, with the MRL we now have the opportunity to visualise targets and OARs during treatment delivery which will enable the chance to explore new compromises between tumour control and toxicity in the future.

For the 28% of fractions where the CTV prostate D95% was estimated to be higher for the MRgART compared to C-arm linac treatment, the magnitude of detriment (55.2 Gy vs 57.5 Gy) was likely to have clinical implications. The CHHiP trial demonstrated that 57 Gy in 19 fractions could not be considered non-inferior to 74 Gy in 37 fractions (itself non-inferior to 60 Gy in 20 fractions) so a detriment of 2.3 Gy in 20 fractions may be enough to compromise biochemical control [22].

For one fraction the clinical MRgART failed to meet the mandatory rectum V60.8 Gy clinical goal. Retrospective analysis of this fraction showed that this clinical goal would have been achieved had an ATP been performed based on the verification image registration (right branch of Fig. 1), highlighting the need to perform the ATP when appropriate. This analysis also demonstrates that radiotherapy performed on a C-arm linac offers a good solution for patients who present with favourable anatomy at the time of reference imaging and demonstrate stable anatomy throughout the course of their treatment. Indeed, if case 3 is excluded from the analysis 98% of mandatory clinical goals were achieved by the MRgART and C-arm linac delivered dose estimates. However, we cannot currently predict which patients may benefit from MRgART based on the reference image, meaning that if more profound hypofractionation for prostate radiotherapy is to be realised, MRgART using the MRL may be the most appropriate treatment technique.

We have observed proportions of the rectum and bladder receiving low doses for MRgART compared to C-arm linac fractional dose estimates. This is due to two main reasons. Firstly, the MRL is only currently able to deliver fixed-field IMRT treatments, compared to the VMAT technique used for the backup plans. Secondly, as part of the decision to implement an MRgART workflow using ATS, we did not attempt to achieve the lowest possible OAR dose in our reference plans as when this plan is propagated to the daily image for adaptive re-planning excessive OAR sparing on the daily geometry can result in unnecessary target compromise. This can be because either the daily anatomy is less favourable than on the reference image or, given that online planning has to occur in a timely fashion, the online optimisation does not have enough time to achieve full target coverage at the lowest achievable OAR dose. Therefore, this study compares the estimates of the MRL and daily IGRT C-arm treatment planning strategies that have been implemented in our department and does not represent the best dose distributions that can be achieved. This study describes our first implementation of MRgART using an MRL and as more patients are treated we aim to refine and improve on the planning strategy. It is hoped that with enhanced delivery capabilities on the MRL (such as VMAT delivery), faster online optimisation allowing OARs to be optimised to a lower dose without compromising targets, and with the opportunity to reduce CTV to PTV margins with MRgART, we should be able to achieve lower OAR dose using the MRL, when compared to a C-arm linac.

Alongside affording CTV to PTV margin reduction due to improved target visualisation and image registration (which would, given the currently-implemented workflow, necessitate more ATP of ATS plans) and the opportunity to deliver more profound hypofractionated treatment regimes, MRgART allows the possibility in the future to deliver a boost dose to the daily position of the dominant lesion visible on MR as well as enabling real-time soft-tissue tracking to account for intra-fraction motion, making MRgART using the MRL an attractive option for prostate cancer treatment going forwards.

## 5 Conclusions

Prostate MRgART is feasible to deliver using a high field MR-linac with the full daily adaptive re-planning workflow. When using standard CTV to PTV margins, radiotherapy performed on a C-arm linac offers a good solution for prostate cancer patients who present with favourable anatomy at the time of reference imaging and demonstrate stable anatomy throughout the course of their treatment. For patients with organs at risk abutting target volumes on their reference image which would necessitate target coverage compromise using a conventional non-adaptive radiotherapy technique, we have demonstrated the potential for target dose coverage improvement for MRgART on an MRL compared to C-arm linac daily IGRT treatment. The ability to adapt treatments to patient anatomy immediately prior to treatment delivery makes MRgART an attractive modality in the future for more profound hypofractionation treatment for prostate cancer.

## Declaration of Competing Interest

The authors declare that they have no known competing financial interests or personal relationships that could have appeared to influence the work reported in this paper.
