# Supplementary material for: Daily adaptive radiotherapy for patients with prostate cancer using a high field MR-linac: Initial clinical experiences and assessment of delivered doses compared to a C-arm linac
Source: Clin Transl Radiat Oncol. 2020 Apr 29;23:35–42. doi: 10.1016/j.ctro.2020.04.011 (PMC7210377; doi:10.1016/j.ctro.2020.04.011)
Supplement: Supplementary data 1 [file mmc1.docx]

**Supplementary data**

**Table S1: CTV to PTV margins, OAR constraints, and target coverage requirements for the PRISM trial.**

| **OAR** | **Dose (Gy)** | **Max Volume (% or cc)** | |
| --- | --- | --- | --- |
|  |  | Optimal | Mandatory |
| Rectum | 24.4 | 80% |  |
|  | 32.4 | 65% |  |
|  | 40.5 | 50% | 60% |
|  | 48.6 | 35% | 50% |
|  | 52.7 |  | 30% |
|  | 56.8 |  | 15% |
|  | 60.8 | 3% | 5% |
| Bladder | 40.5 | 50% |  |
|  | 48.7 | 25% |  |
|  | 52.7 |  | 50% |
|  | 56.8 | 5% | 35% |
|  | 60.8 | 3% | 25% |
| Bowel | 36.5 | 78cc | 158cc |
|  | 40.5 | 17cc | 110cc |
|  | 44.6 | 14cc | 28cc |
|  | 48.7 | 0.5cc | 6cc |
|  | 52.7 |  | <0.01cc |
| Penile Bulb | 40.5 |  | 50% |
| **PTV** | **Volume (%)** | **Minimum dose (Gy)** | |
|  |  | Optimal | Mandatory |
| PTV_6000  *5 mm left, right, ant, superior, inferior, and 3 mm post expansion of ‘prostate CTV’* | 95 |  | 57.0 |
|  | 98 | 57.0 | 55.8 |
| PTV_4860  *5 mm isotropic expansion of the combined prostate and SV CTVs* | 95 |  | 46.2 |
|  | 98 | 46.2 | 45.2 |
